# Medical misogyny: understanding epistemic injustice to achieve safer healthcare for women in the UK

**DOI:** 10.1093/medlaw/fwag009

**Published:** 2026-04-18

**Authors:** Laura O’Donovan, Sarah Devaney, Alexandra Mullock, Victoria L Moore

**Affiliations:** School of Law, University of Sheffield, Bartolome House, Winter Street, Sheffield, S3 7ND, United Kingdom; Law Department, University of Manchester, Williamson Building, Oxford Road, Manchester, M13 9PL,United Kingdom; Law Department, University of Manchester, Williamson Building, Oxford Road, Manchester, M13 9PL,United Kingdom; Law Department, University of Manchester, Williamson Building, Oxford Road, Manchester, M13 9PL,United Kingdom

## Abstract

Since 2019, numerous reports (both official and charity-led) have been published detailing patient care and safety failings in areas of women’s healthcare in hospitals across the UK. A common theme that has emerged from these reports is a sense that the voices of women and people seeking maternity care and/or treatment for female health conditions are frequently dismissed and silenced. While many of the examples detailed in these reports have been appropriately recognized as both individual and systemic failings in patient care, here we apply Miranda Fricker’s epistemic injustice framework to these issues. We argue that testimonial injustice (a form of discriminatory epistemic injustice) forms a core part of the experience of some of these patients’ care resulting in a compounding of their experience of harm. Despite various exhortations within the reports that women should be listened to, the evidence demonstrates that women’s testimonial knowledge is systematically devalued. We thus contend that a fundamental reframing of the issue is required and that understanding how and why epistemic injustice occurs is critical to developing a better understanding of how to avoid it, both in the provision of women’s healthcare and in its regulation.

## I. Introduction

Over the past 6 years, numerous reports have been published detailing the various avoidable harms suffered by women and people assigned female at birth as a result of patient care and safety failings in areas of women’s healthcare^1^ in hospitals across the UK. Here we refer to healthcare services that focus on the treatment and care of diseases and conditions that are unique to people of female biology, predominantly women. These concern, for example, uterus and ovary health, cervix, vagina and vulva health, and pregnancy.^2^ In this article, we argue that many of the examples of poor care highlighted by the different reports can and should be viewed as instances of epistemic injustice, causing harm that is avoidable, and that understanding epistemic injustice is an important step towards ameliorating medical misogyny and its resulting physical and emotional consequences.

We focus on seven high-profile reports published between 2019 and 2024 concerning areas of ‘women’s healthcare’ rather than investigations into healthcare across the National Health Service (NHS) more broadly. This stems from a recognition of the documented gender disparities in healthcare provision and outcomes, as highlighted in the previous UK government’s Women’s Health Strategy.^3^ There it was noted that ‘[n]ot enough focus is placed on women-specific issues like miscarriage or menopause’,^4^ and that ‘too often it is women whom the healthcare system fails to keep safe and fails to listen to’.^5^ Given this, a targeted examination of areas where these issues may be most pronounced is required. The reports we draw from are the 2020 Cumberlege Report^6^; four reports published in 2022: the Ockenden Report,^7^ the Birthrights Report,^8^ the Five X More Report,^9^ and the Kirkup Report^10^; the 2023 Unheard Report^11^; and the 2024 Birth Trauma Report.^12^ While the focus and scope of each of these reports is different, they collectively identify multiple ways in which women and people with female anatomy have suffered myriad harms either (i) as part of their maternity care, (ii) when receiving care for conditions affecting their female anatomy, and/or (iii) during the course of other healthcare treatment which has then affected elements of their reproductive health and well-being.

A common theme that we argue has emerged in the findings of these inquiries is a sense that the voices of women and people seeking care for female health conditions are frequently dismissed and silenced.^13^ While many of the examples and cases detailed in these reports have been appropriately recognized as both individual and systemic failings in patient care, in this article, we apply Miranda Fricker’s theory of epistemic injustice to further elucidate these issues. We suggest that this, in turn, can contribute to the implementation of more effective regulatory regimes to prevent harm and avoid compounding it in its aftermath.^14^

We argue that testimonial injustice (a form of discriminatory epistemic injustice) formed a core part of the experience of some patients’ care, compounding their experiences of their illness or condition, and/or of their experience of harm caused during their treatment. In some instances, ignoring or dismissing patient concerns resulted in diagnostic failings and negligent treatment, leading to serious harm and even death.^15^ It is therefore critical to examine how and why epistemic injustice occurs in order to develop a better understanding of how to avoid it, both in the provision of care and in its regulation.

In 2022, the Conservative government’s response to failings in women’s healthcare was to launch a Women’s Health Strategy for England^16^—an initiative that the current Labour government has explicitly committed to take forward as part of its 10-year plan to fix the NHS.^17^ The aim of the strategy is: (i) to boost health outcomes for women and girls and (ii) to radically improve the way in which the health and care system engages and listens to women and girls.^18^ It is notable that within this strategy, improving how healthcare professionals (HCPs) and the wider healthcare system centres and respects women’s voices constitutes one of the two key policy aims. What this suggests, and what we hope to demonstrate in this article, is that testimonial injustice experienced by women is both a widespread and pernicious issue, amounting to ‘medical misogyny’^19^ that requires a much more robust regulatory response.

We build our case as follows. In Section II, we provide the context by first briefly setting out the nature and scope of the inquiries into patient care and safety failings that are our primary focus. Section III outlines the account of epistemic injustice which underpins our analysis, and its manifestation in the healthcare context. In Section IV, we evidence the ways in which some of the individual and structural identity prejudices operating in women’s healthcare have led to testimonial injustice. Section V explores and articulates the primary and consequential harms patients have suffered as a result of these injustices. We also consider how testimonial injustice damages trust in healthcare systems and professionals, which in turn impairs the therapeutic relationship. On the basis of this evidence, in Section VI we provide recommendations as to how patient safety regulation frameworks need to improve, both to provide accurate insights into where and why failings are happening, and to help avoid perpetuating the harms we have identified.

Finally, we wish to emphasize that while our analysis focuses on reports concerning reproductive and sex-specific healthcare, the patterns of epistemic injustice we identify are likely not confined to these domains of women’s healthcare. The choice to focus here reflects the availability of detailed inquiry reports rather than a judgment that these are the only areas affected by testimonial injustice.

## II. Background and methodology

As mentioned in the introduction, in this article, we focus specifically on seven high-profile reports published between 2019 and 2024 concerning areas of ‘women’s healthcare’. There is now a long and well-established history in the UK of extensive reviews of, and inquiries into, patient safety failings, identifying problematic cultures of practice, including failures to listen to patients, and delays in taking action to improve patient care.^20^ Despite the resulting recommendations in review and inquiry reports on how to improve care, patients have continued to suffer appalling failings in treatment provision. See Table 1 for a chronological overview of the seven reports we focus on in this article.

**Table 1:** Overview of reports.

**Report title**	**Year of publication**	Type of investigation	Period of care covered	Area of healthcare	**Key findings** (emphasis added)
Report of the Independent Medicines and Medical Devices Safety Review (‘the Cumberlege Report’)	July 2020	Independent review commissioned in 2018 by the Secretary of State for Health and Social Care	Cases spanning from the 1960s (Primodos first used) to the time of review (exact dates unspecified)	Hormone pregnancy tests; Sodium valproate (an anti-epileptic drug); and Pelvic mesh implants	Across all three health interventions, common themes were: The lack of information to make informed choices; Lack of awareness of who to complain to and how to report adverse events; **The struggle to be heard;** **Not being believed;** **Dismissive and unhelpful attitudes on the part of some clinicians;** **A sense of abandonment;** **Life-changing consequences, not only for those directly affected, but for their families and friends too;** Breakdown of family life; Loss of jobs, financial support and sometimes housing; Loss of identity and self-worth; A persistent feeling of guilt; Children becoming their mothers’ and siblings’ carers; Clinicians are untutored in the skills they need to make a proper diagnosis; **Clinicians not knowing how to learn from patients;** Inaccurate or altered patient records; and A lack of interest in, and an inability to deliver, the monitoring of adverse outcomes and long-term follow-up across the healthcare system.
Final Report from the Independent Review of Maternity Services at The Shrewsbury and Telford Hospital NHS Trust (the ‘Ockenden Report’)	March 2022	Independent review commissioned in 2017 by the Secretary of State for Health and Social Care	2000–2019	Maternity services	Patterns of repeated poor care, including: ○ **Failures to investigate, inform, and listen;** ○ **Care that was not in line with best practice at the time;** ○ **Care that could have been significantly improved;** ○ Repeated failures to escalate concerns in both antenatal and postnatal environments; ○ Delays in treatment; and ○ Lack of appropriate escalation; Multiple failures in governance and leadership with devastating consequences: ○ Failure to follow national clinical guidelines; ○ Lack of action from senior clinicians; ○ Lack of psychological safety in the workplace and cultural issues; ○ Lack of compassion; ○ Significant staffing and training gaps; ○ Unsafe inpatient-to-staffing ratios; and ○ Lack of oversight and full understanding by the Trust board.
Inquiry into Racial Injustice and Human Rights in UK Maternity Care (‘the Birthrights Report’)	May 2022	Inquiry by charity	Poll—last 5 years = 2017–2022 Oral evidence—21 February to 22 January	Maternity services	Common themes emerged from the evidence: Lack of physical and psychological safety; **Being ignored and disbelieved;** **Racism by caregivers;** **Dehumanization;** Lack of choice, consent, and coercion; Structural barriers; and Workforce representation and culture.
The Black Maternity Experiences Survey (the Five X More Report)	May 2022	Study by a charity	2019–2021	Maternity services	Negative experiences were found to fit within a framework overarched by three interrelated constructs centred around the healthcare professional (HCP): **Attitudes (eg using offensive and racially discriminatory language; being dismissive of concerns);** Knowledge (eg poor understanding of the anatomy and physiology of Black women; poor understanding of the clinical presentation of conditions in babies of Black women); and **Assumptions (eg racially based assumptions about the pain tolerance, education level, and relationship status of Black women).**
Report of the Independent Investigation into Maternity and Neonatal Services in East Kent (‘the Kirkup Report’)	October 2022	Independent review commissioned in 2020 by Minister of State for Patient Safety, Mental Health and Suicide Prevention, Department of Health and Social Care	2009–2020	Maternity services	The origins of the harm identified lie in the following: Failures of teamworking; Failures of professionalism; **Failures of compassion;** **Failures to listen;** Failures after safety incidents; and Failure in the Trust’s response.
Unheard: Women’s Journey Through Gynaecological Cancer (the Unheard Report’)	December 2023	Inquiry by the Health and Social Care Committee, Welsh Parliament	Not specified. Call for evidence between 22 October and 23 March appears to focus on current rather than historic experiences.	Gynaecological cancer care	Of particular relevance to this article: A strong theme that came through in the evidence we heard on this inquiry was that **many women continue to feel unheard in healthcare settings** (para 31). It is clear that **women’s health needs to be given a higher priority than has previously been the case.** Women need to access the care they need when they need it. The health service needs to be responsive in providing that care. **Most importantly, it needs to listen to women and respond to their health concerns appropriately.** (para 35).
Listen to Mums: Ending the Postcode Lottery on Perinatal Care (‘the Birth Trauma Report’).	May 2024	Inquiry by All-Party Parliamentary Group on Birth Trauma	Open ended	Maternity services	Key themes: **Failure to listen;** Lack of informed consent; **Poor communication;** **Lack of pain relief;** **Lack of kindness;** Breastfeeding problems; Poor post-natal care; The impact of covid and Complaints and medical negligence.

Our selection criteria focused on reports addressing various aspects of women’s health, with particular attention to those that garnered significant public and policy attention. While not employing a systematic review methodology, we prioritized reports that demonstrated substantial impact within the broader discourse on the gender health gap. This approach allowed for the identification of key documents that have shaped contemporary understanding of women’s health disparities and associated policy responses.

Our analytical framework employed Fricker’s conceptualization of testimonial injustice to examine the selected reports. We specifically sought to identify instances where women’s testimonies and experiences were subject to credibility deficits due to prejudicial assumptions. The analysis focused on identifying patterns of testimonial injustice, particularly examining contexts where women reported feeling unheard, dismissed, or experiencing systematic undermining of their credibility within healthcare settings. Through this theoretical lens, we extracted and analysed quotations that exemplified these dynamics, seeking to illuminate the epistemic dimensions of women’s healthcare experiences.

## III. Understanding epistemic injustice

Long before the philosopher Miranda Fricker coined the term epistemic injustice to describe what she suggests is a ‘wrong done to someone *specifically in their capacity as a knower*’,^21^ Black feminist activists and writers such as Anna Julia Cooper and Sojourner Truth were sharing their experiences of having their truth silenced and dismissed.^22^ Indeed, examples of epistemic resistance (calling attention to the ways in which particular knowers have been silenced and marginalized)^23^ ‘can be traced to [historical] discussions around intersections of power, knowledge and oppression’.^24^ In 1867, for example, Sojourner Truth, a woman’s rights activist and abolitionist, spoke of society’s silencing and denying the credibility of Black women as knowers.^25^ In recent times, the concept of epistemic injustice has gained prominence through the work of Fricker, who identifies two forms of injustice that are distinctly epistemic in nature: testimonial injustice and hermeneutical injustice.^26^

### A. The central cases of testimonial and hermeneutical injustice

Testimonial injustice occurs when, owing to some particular prejudice—the ‘ethical poison’ of testimonial injustice^27^—a hearer unduly attributes a deflated level of credibility to a speaker, which affects their acceptance of the speaker’s testimony, and results in them being designated as an epistemic lesser.^28^ It is, according to Fricker, the wrongful undermining of the speaker specifically in their capacity as a knower, which may lead to the hearer missing out on knowledge as a result.^29^ This may occur after a speaker has told or asserted something to the hearer, asked a question, or contributed to an epistemic exchange in some other way. It may also operate pre-emptively,^30^ when the hearer fails to even solicit the views of the epistemic subject because they perceive them to lack credibility.^31^

By contrast, hermeneutical injustice occurs where a particular type of epistemic gap exists between a person’s experience, their ability to render that experience communicatively intelligible and the hearer’s ability to understand it.^32^ This lack of sufficient shared concepts between subjects is owed to what Fricker terms ‘hermeneutical marginalisation’—unequal participation in the exercise of meaning-making practices.^33^ The example frequently used to illustrate it is that of sexual harassment.^34^ Prior to society’s collective understanding and labelling of this social experience as sexual harassment, no term to describe this concept existed due to the fact that women’s experiences were not adequately reflected by common shared concepts.^35^ As such, those experiencing this kind of abuse may have struggled both to make themselves understood when trying to share their experience and to be believed by others due to the previous paucity of the shared concept.^36^

Our focus in this article is on the effect of testimonial injustice in women’s healthcare.^37^ While prejudice can take a variety of different forms, prejudice in the central case of testimonial injustice advanced by Fricker is that of ‘identity prejudice’—‘a label for prejudices against people *qua* social type’.^38^ Typical examples include, but are not limited to, negative social stereotypes or biases on the basis of gender, race, sex, religion, socio-economic status, or educational status. Fricker draws on the fictional example of the case of Tom Robinson’s jury trial in Harper Lee’s novel *To Kill a Mockingbird* to illustrate an (extreme) example of testimonial injustice.^39^ Here, we highlight what we suggest is an illustrative example from the healthcare setting.^40^

Kathy had been experiencing heavy periods that left her feeling so faint that she was unable to stand upright. Four different medical professionals suggested to her that she was struggling with anxiety and not a physical illness. According to Kathy, her primary care doctor told her that ‘all your symptoms are your [sic] imagination’.^41^ It was only when Kathy pushed for an ultrasound scan after suffering with debilitating symptoms for 9 months that she was diagnosed with uterine fibroids requiring surgical intervention.^42^ She was suffering from anaemia and not anxiety.^43^ This example arguably bears all the hallmarks of testimonial injustice. First, the treating HCPs afforded Kathy reduced credibility as a knower of her own bodily experiences, dismissing her accounts of physical symptoms. Secondly, this credibility deficit appears rooted in prejudicial stereotyping of ill women as anxious, overly emotional, and unreliable narrators of their experiences. Thirdly, this epistemic wrong caused significant harm: Kathy’s health concerns were dismissed, and the opportunity for early diagnosis and treatment was missed, prolonging her suffering for 9 months. These features taken together constitute a clear case of testimonial injustice, and unfortunately, as we demonstrate in Section IV, Kathy’s experience is not an isolated one.

### B. The harms of epistemic injustice

The harms of the above types of epistemic injustice are characterized by Fricker as primary (intrinsic) and secondary (extrinsic) harms.^44^ The primary harm of testimonial injustice is that one is undermined in one’s capacity as an epistemic subject.^45^ This is to say that one is ‘undermined or otherwise wronged in a capacity that is essential to human value’,^46^ that is, as a knower and therefore a credible speaker.^47^ The secondary harms are the practical, often tangible consequences which flow from the primary harm, and we therefore describe these as ‘consequential harms’.

Consequential, secondary harms consist of ‘a range of possible follow‐on disadvantages, extrinsic to the primary injustice in that they are caused by it rather than being a proper part of it’.^48^ The first category of secondary harm is the *practical* consequences that may follow, which in healthcare may be significant physical and/or psychiatric harm in circumstances where it is the failure of care, that is, ignoring or dismissing the patient’s testimony, that led to the harm occurring. As we discuss in Section , such tangible harm might also be regarded as a legally recognizable harm invoking civil or even (rarely) criminal liability. The second category of consequential harm is ‘(more purely) epistemic harm’,^49^ such as the target of the testimonial injustice becoming more widely perceived as having lower epistemic status, or their own confidence in their epistemic capacity being eroded into the future.^50^ Women who experience repeated dismissal of their symptoms may internalize the message that their accounts are unreliable. This loss of confidence can in turn damage trust in healthcare professionals and systems, with evidence suggesting that some women become so traumatized by experiences of dismissal that they will not seek care unless it is absolutely life-threatening.^51^ We return to the consequences of this erosion of trust in the Section V.

### C. Epistemic injustice in the healthcare context broadly

A wide range of literature exists exploring the occurrence of epistemic injustice due to the power imbalance, which potentially makes patients vulnerable in a number of health contexts.^52^ Carel and Kidd, for instance, explain that healthcare systems are grounded upon ‘complex structures of epistemic norms and expectations, both implicit and explicit that create knowledge asymmetries’,^53^ for example, the privileging of certain forms of knowledge. In the medical context, they suggest this refers to knowledge gained from medical training and theory rather than that which is grounded in patients’ embodied experience of illness.^54^ They argue that the power structures inherent in healthcare institutions and systems ‘can also indirectly affect the epistemic confidence and capacities of ill persons’,^55^ in that illness may render patients vulnerable and fragile in ways that ‘challenge the autonomy and dignity that epistemic agency requires’.^56^ As a result, it is suggested that ill persons are particularly vulnerable to both testimonial and hermeneutical injustice.^57^ This arises for the following two reasons.

First, in the clinical context, patients find themselves within a particular power relationship, for ‘power is present in all interpersonal relationships; there is thus no interaction in which power is not relevant in healthcare’.^58^ While patient empowerment and moving away from medical paternalism have been an important health policy within the NHS^59^ (and other healthcare systems),^60^ by default, it is always HCPs who ‘have power available to them through their cultural and symbolic capital legitimized by the institution of medicine’.^61^ As such, HCPs, in particular, physicians, enjoy epistemic privilege or advantage in ways that may be warranted by virtue of their education and training,^62^ and also unwarranted where, for example, such advantage is grounded in presumptive judgements about the epistemic credibility and thus privilege that should be afforded to someone.^63^

Secondly, negative stereotypes and biases applicable to patients and ill persons as a social group often diminish their perceived epistemic competence and capabilities.^64^ The ill persons that Kidd and Carel are referring to are those who experience chronic somatic conditions^65^—a specific subgroup of ill patients.^66^ However, the stereotypes of chronically ill persons they identify are stereotypes that are applicable to the category of ill persons or patients in the healthcare setting generally, though chronically ill persons may be subjected to them more frequently and possibly to a greater degree. These stereotypes are particular identity characterizations unique to the clinical context,^67^ including descriptions of ill persons as ‘cognitively impaired or emotionally compromised’,^68^ or irrational, anxious or ‘psychologically dominated by their illness in a way that warps their capacity to accurately describe and report their experiences’.^69^ While birthing women and people are generally not a group who are ‘ill’, given pregnancy is not a disease, as one of us has noted, their engagement with maternity services positions them within the healthcare system’s hierarchical power structure, which tends to afford greater epistemic privilege to HCPs at the expense of affording sufficient credibility to the lived experience of patients.^70^ As such, she posits that just as with women engaging with the healthcare system as ‘patients’, birthing women and people may also be vulnerable to epistemic injustice as female users of the healthcare system.^71^

Such prejudicial characterizations of patients, ill persons, or birthing women and people may be further compounded by other social prejudices^72^ held by HCPs, which, according to Kidd and Carel, tend to ‘prejudicially deprive ill persons of the prerequisites of reliable epistemic conduct … and also attribute to them characteristics … that are usually (and contentiously) supposed to be opposed to rationality and reliability’,^73^ leading to their testimonies being accorded a lower level of credibility than they otherwise would have been. Having outlined the account of epistemic injustice underpinning our analysis and an account of its operation in the healthcare context broadly, we now apply this to the case of women’s healthcare.

## IV. Evidencing testimonial injustice in women’s healthcare

Drawing on the reports outlined in Table 1, in this section, we evidence the existence of testimonial injustice in women’s healthcare in the UK. Here, we argue that women’s adverse and sometimes catastrophic health experiences are, in part, a consequence of prejudices against them as a social group. Indeed, taken together, the reports demonstrate a pernicious systemic failure in the provision of women’s healthcare in the NHS that is reflective of a wider healthcare culture in which female bodies are mythologized and accounts of female pain are distrusted.^74^ Below, we consider two prejudices evident in the reports considered: gender and race.

### A. Gender prejudice

Inequality has long existed between men and women. As Auchmuty posits, ‘women have not simply been unequal to men, they have been *oppressed* by them and the institutions of patriarchy constructed to perpetuate male power’.^75^ Such oppression and division are also reproduced in the medical sphere, and Cleghorn, for example, argues that ‘at every stage in its long history, medicine has absorbed and enforced socially constructed gender divisions … [which] have traditionally ascribed power and dominance to men’.^76^ This is because although medicine and its history may appear to be objective, logical, and based on evidence, it too ‘is every bit as social and cultural as it is scientific’.^77^ Medicine has developed within these conditions and, as such, ‘male dominance –and with it the superiority of the male body– was cemented into medicine’s very foundations’.^78^

As the concept of modern medicine emerged from Ancient Greece, so too did the idea that the female body was inferior to the male body. Texts from the Presocratics to Galen typically positioned the male body as *the* point of reference from which the female body deviated.^79^ Females were thus characterized as incomplete or the inverse of the male body.^80^ Biological difference not only defined the female body but also constrained it, with ‘women’s illnesses and diseases consistently related back to the “secrets” and “curiosities” of her reproductive organs’.^81^ This can be seen in the emergence and history of the condition ‘hysteria’ (from the Greek *hystera*, or uterus), the cause of which the Ancient Greeks and the Victorians attributed to the uterus.^82^ Notably, it was not until 1980 that ‘hysterical neurosis’ was removed from the Diagnostic and Statistical Manual of Mental Disorders (DSM-III).^83^

The practice of medicine and the medical establishment have thus evolved and developed in the context of particular beliefs and myths about the female body, and patriarchal attitudes within societies where historically women were effectively owned by their father or husband.^84^ Medical knowledge and research has also been shaped by these notions. For example, clinical studies have historically excluded female participants because of the ‘risks’ posed by hormones or pregnancy. Instead, research data collected from males has been generalized to females and intersex people.^85^ This has resulted in a dearth of knowledge when it comes to how different diseases present and manifest in women and those with female biology, and also, an extant knowledge gap about conditions and diseases which specifically affect female reproductive anatomy—the uterus, ovaries, and fallopian tubes.^86^

Within the seven reports under consideration, specifically in the testimonies of those who provided evidence, there is a sense that women were not seen as experts of their own bodies, conditions, or symptoms. Consider, for example, the experiences of two former mesh implant patients:

‘They would tell you there is nothing wrong with you and that you are just a hysterical woman…’ – Teresa Hughes, Meshies United.^87^

‘So I went back to the consultant to discuss things. Unfortunately he is very pro mesh and when I asked if he thought my issues were linked to my TVT-O^88^ he actually screamed at me “you need to stop listening to the media and those bloody women, I fit hundreds of these every year and you’re the only person I’ve seen who is complaining and thinking you have problems”.’^89^

A gynaecological cancer patient also felt disbelieved when she first reported her symptoms, but was later diagnosed with uterine leiomyosarcoma:

Primary care and my experience with my GP was [ ], disappointing, to say the least; so many phone calls, me chasing, being dismissed. I called it medical gaslighting by the end, and I think the reason I ended up in tears with the final GP appointment was finally feeling vindicated, like I’m not a neurotic woman who’s making a fuss over nothing, which is definitely how I was made to feel.^90^

Similarly, a woman who had experienced traumatic birth complications had her symptoms initially wrongly dismissed as psychological:

One woman who was in extreme pain for the last few weeks of her pregnancy, had “anxious mother” recorded on her notes. In fact, she was bleeding internally as the result of spontaneous hemoperitoneum, a rare and often fatal complication of pregnancy whereby tissue had torn behind her uterus.^91^

A woman who experienced severe pain during labour was also dismissed without her concerns being taken seriously:

“I know I haven’t had a baby before but this is my body and I know what’s going on, and this doesn’t feel right, this doesn’t feel safe. I was expecting to be in pain, I’m not stupid, but this feels unsafe, this amount of pain; and being told, ‘you’ve never had a baby before, I don’t know what you expected”.^92^

And a woman who experienced a missed recto vaginal fistula, causing personal care issues, was dismissed without her concerns being validated:

After examining me the doctor informed me that I’d had a large baby and that had caused in her words “a baggy fanny”. To say I was upset is an understatement and despite telling her that I could tell the wind was coming from my back passage and passing through to the front, she said no further investigation was required.^93^

These testimonies evidence some of the gendered tropes and negative stereotypes that may impact the care provided by some HCPs; tropes that might characterize women as: anxious^94^; overly emotional, irrational, or hysterical^95^; dramatic and prone to over-reaction^96^; precious^97^; and the cultural belief that women’s bodies, particularly Black women’s bodies, are built to experience and withstand a greater degree of pain.^98^ All patients quoted here arguably received a deflated level of credibility from their treating HCP than they otherwise would have, such that their testimonies were presumed to be unreliable and irrelevant. Each experienced testimonial injustice, which is suggestive of bias and prejudice against them *as women*.

Indeed, framing the women as ‘hysterical’, ‘anxious’, ‘overreacting’, or the inference that their account of their symptoms was the product of some kind of ‘mass hysteria’ allowed the HCPs to *explain away* the symptoms the women sought help for. As we noted above, hysteria was once a common diagnosis for a variety of symptoms that women experienced (including those that we would now recognize as depression, anxiety, post-traumatic stress disorder, menopause, epilepsy, or infertility).^99^ And while hysterical neurosis no longer exists as a recognized diagnosis, it appears to be hard to escape the long shadow of women being cast as hysterical, emotional, and anxious, and the mischaracterization as psychological of the very real and physical problems they report. Gender stereotypes and biases, whether consciously or unconsciously held by the relevant HCPs in these examples, played a part in shaping the judgements they made about patients and the reliability of their testimonies, and the extent to which their patients were truly *listened to*.

### B. Racism

In addition to gender, cultural biases, stereotypes, and racism together also function as one of the forms of identity prejudice underlying testimonial injustice in women’s healthcare.^100^ Identity is not one-dimensional and a person’s experience will be shaped by the way in which different factors intersect within broader power relationships and structures to affect access to and experiences of healthcare.^101^ In the UK, Black women are at 2.3 times greater risk of maternal mortality than White women, while Asian women have a risk of 1.3 times that of White women.^102^ Knight et al. reviewed the clinical records of 54 women in the UK who died during or up to a year after the end of pregnancy between 2009 and 2018, and found numerous health care system factors which they identified as ‘structural biases’ in the care provided.^103^ The biases were found to affect women from all ethnic groups who had ‘complex health and social problems’;^104^ nevertheless, they also identified biases affecting different ethnic groups more severely. Black women were particularly affected by a lack of individualized and nuanced care,^105^ and this was reflected in instances of testimonial injustice noted in the reports and evidence we considered.

For example, a Black woman’s concerns about her foetus were denied and dismissed for hours, which ended up with her requiring surgery:

“I ended up having to go into surgery because my daughter’s heartbeat had started to drop…I feel like we only got to this stage because the first midwife had dismissed me for hours when I was trying to…tell her that something is wrong.” (Black woman)^106^

Pain management requests are also an area in which the global majority of women are subjected to prejudice on the basis of their ethnicity, with biased views about their supposed high or low tolerance levels for pain leading to the same outcome—insufficient provision of pain relief. One Black woman who provided evidence to the Birthrights inquiry was told ‘women like you’ do not need pain relief and was advised not to make so much noise.^107^ Conversely, many Asian women, particularly those of South-Asian heritage, referenced the ‘princess’ stereotype, and HCPs reported that women from these backgrounds were perceived as ‘precious’, less able to tolerate pain, and often ‘made a fuss’.^108^ Such prejudice in the area of pain relief is typified in the following examples:

A Black woman who was struggling to breathe was dismissed and disbelieved when she reported her symptoms, but was later diagnosed with a pulmonary embolism:

I was literally about to go home, there was a genuine 10 minute period between me going home with a potential embolism [and] it was just so weird, like the day before I remember constantly saying “my chest feels really tight, I can’t breathe, I can’t stand, I can’t walk, I can’t do anything”. I had to get a bed pan to pee in because I couldn’t stand for long without losing my breath and I’m like, you know I get it, I was anxious. But also, I genuinely couldn’t breathe, you know, and that’s the moment that always scares me because you hear so many of these stories, especially from Black women where their pain hasn’t been taken seriously during this crucial moment. […] My pain wasn’t taken seriously and I was dismissed to the point that could have actually, you know, cost me my life.^109^

Other Black women reporting pain symptoms had their concerns minimized and written off:

One Black mixed woman was told that she was being “*dramatic*” during administration of an epidural which subsequently resulted in numbness in her leg for the following three months.^110^

Women reported often that their symptoms were minid and that their pain or worries about their baby were not taken seriously. For instance, one Black woman was told that she was *“being loud for no reason”*.^111^

Similarly, a global majority woman had concerns about her baby dismissed, which she felt was due, in part, to her race:

“During the postnatal stage, I was telling the nurses she wasn’t latching and was bringing up some yellow fluid. I was dismissed at first and then they assessed and took her to neonatal. There were too many incidents of not being taken seriously and my worries not being validated. I feel race probably played a part as I am a turban-wearing Sikh so look very different.”.^112^

The above accounts illustrate how racial prejudice can lead to testimonial injustice. Again, all patients quoted here arguably received a deflated level of credibility from their treating HCP with their testimonies being reinterpreted through racialized stereotypes rather than being believed in the first instance. Complaints about pain, for example, were reframed as ‘being dramatic’ or ‘loud’—exemplifying the stereotype or trope of the ‘Angry Black Woman’^113^ and undermining the legitimacy of women’s self-report of pain. Similarly, the Sikh woman’s reflection that her visible religious identity probably contributed to her concerns being dismissed underscores how being perceived as *other* can exacerbate credibility deficits. These examples also illustrate how intersecting racial and gender prejudices place ethnic minority women at more acute risk of their testimonies being dismissed and disbelieved.

The House of Commons Women and Equalities Committee has rightly stated that

[t]he causes of the appalling disparity in maternal deaths are multiple, complex and still not fully understood. Fixating on any one cause risks over-simplifying the problem and placing blame on the very women who are most at risk.^114^

Nevertheless, they, and others, have used the evidence provided by and on behalf of minority ethnic women about the failings in their healthcare to identify one of the bases for these failings and resulting harms as racism.^115^ While more needs to be done to explore and understand the causes and nature of this prejudice,^116^ the examples of care highlighted in recent reports demonstrate how racism in healthcare is embedded in the social and institutional structures of healthcare. Addressing these disparities requires more than attention to isolated care incidents; rather, it demands a critical examination of the organizational and cultural factors that consistently undermine the credibility of certain patients and perpetuate unequal treatment.^117^

### C. Testimonial injustice as a form of medical misogyny

Having evidenced above the operation of gender and racial prejudice as distinct but intersecting drivers of testimonial injustice, we now consider how, taken together, they amount to a systemic phenomenon that we characterize as medical misogyny. In the context of women’s healthcare, whether women verbalize their distress and concerns to their HCPs, or there are clinical signs and symptoms which, in theory, provide a more objective and disinterested account of their condition, their narratives tell us that there is something stopping those involved in their care from not only *hearing*, but also actively *listening* to them. This, we suggest, is a form of medical misogyny. We distinguish here between hearing as a passive activity and listening, combining a taking in of information *and* understanding of meaning, together with the propensity to believe and be willing to act on it. The distress this can give rise to was noted in the Cumberlege report:

[P]atients–almost universally women–spoke in disbelief, sadness and anger about the manner in which they were treated by the clinicians they had reached out to for help. The words ‘defensive’, ‘dismissive’ and ‘arrogant’, cropped up with alarming frequency. They spoke of being ‘gaslighted’ and of not being believed, particularly in relation to pelvic mesh and the suffering of pain.^118^

Far from being an isolated problem with a few ‘bad apple’ HCPs, or a problem occurring in just one or two hospitals, the reports show that these care failings are endemic across the system of women’s healthcare, from research, to diagnostics, to care provision. In relation to maternity services in particular, the scale of patient safety failings was acknowledged by the Secretary of State for Health and Social Care, Wes Streeting, during an address at the Royal College of Obstetricians and Gynaecologists World Congress in June 2025. Announcing the launch of a national investigation into maternity and neonatal services, Streeting stated:

we have to acknowledge that this has become systemic. It’s not just a few bad units up and down the country. Maternity units are failing. Hospitals are failing. Trusts are failing. Regulators are failing. There’s too much obfuscation, too much passing the buck and giving lip service, too much shrugging at a cultural problem that we fail to address.^119^

The evidence of testimonial injustice in women’s healthcare in the NHS is further illustrated by data published by the UK government in 2022, which informed the first Women’s Health Strategy for England.^120^ For example, in response to the call for evidence to inform the Strategy, 84 per cent of nearly 100,000 survey participants^121^ reported that ‘there had been instances in which they had not been listened to by healthcare professionals’.^122^ This occurred not just during initial appointments to discuss medical problems, but across the life cycle of the provision of care, including during subsequent appointments, discussions about options for treatment, and follow-up care.^123^ Furthermore, according to a recent report by the Women and Equalities Commission on women’s reproductive health conditions, ‘a common theme in the evidence received was that of women’s pain being dismissed, not just due to a lack of understanding as to what might be causing it but because of a lack of empathy and “medical misogyny”’.^124^

We suggest that testimonial injustice in women’s healthcare exemplifies this medical misogyny in that it embodies the entrenched and systematic devaluation of women’s voices and knowledge. Uncovering and understanding epistemic injustice in the context of women’s healthcare matters precisely because women are wrongfully excluded from contributing knowledge about their own bodies and experiences. This epistemic wrong has direct practical consequences for women’s health: accurate diagnosis and effective treatment depend fundamentally on healthcare professionals understanding patients’ experiences. When women’s testimony is systematically discredited, their symptoms may be misinterpreted, underlying conditions missed, and inappropriate treatments prescribed, leading to prolonged suffering and potentially serious health consequences—as we go on to demonstrate. This not only harms individual women but also impoverishes medical knowledge more broadly by systematically excluding women’s perspectives from healthcare discourse, perpetuating gaps in understanding of women’s health conditions.

## V. Epistemic injustice and patient harm

For patients, the effect of their testimonies being ignored and disregarded may be devastating and the consequences far-reaching.^125^ This includes patients feeling (even more) vulnerable, an increase in the distance between HCP and patient in terms of the structural power relationship, and the preclusion of the possibility of shared decision-making in which the patient takes an active role in their care.^126^ When this type of testimonial harm is perpetrated, it also leads to an erosion of trust, which further threatens the therapeutic relationship. In this section, we consider examples of both primary and secondary harm, which the reports show can occur in women’s healthcare.

### A. The harms of testimonial injustice

The reports discussed provide numerous examples of patients harmed as a result of their testimony being dismissed. The broad problem of primary harm is illustrated here:

This “not being listened to” took several forms. We saw a pattern of women, particularly first-time mothers, being made to feel patronised and demeaned when their concerns were dismissed as overreactions and unnecessary anxieties based on “first-time nerves”. There were women whose concerns about the wellbeing of their unborn babies were ignored; and women on their second or later pregnancies whose personal knowledge, experience and understanding of their own bodies informed their convictions that something was wrong, but whose concerns were either ignored or dismissed. There were also women whose legitimate concerns about their newborn babies were not taken seriously.^127^

Often, when patient concerns are ignored or dismissed in this way, more serious and tangible harm then occurs as a direct consequence. The following example illustrates how the primary harm (being undermined as an epistemic subject) means that crucial information that should have informed the treatment plan was disregarded, often causing (or almost causing) tangible, potentially serious, and even potentially fatal harm (secondary harms):

I started to have a great pain, I felt the induction pain and I was screaming. Unfortunately, the midwife didn’t really believe me, she was like ‘how are you having the pain with you having an epidural?’ She didn’t believe me, she didn’t act as though she believed me and I was having great pain. Then after me screaming and shouting, they asked for the doctor to check my epidural and when he poured cold water on my legs, I felt it.^128^

Other examples of primary harm include patients in early labour whose concerns were dismissed, and whose babies subsequently died.^129^ These cases represent testimonial injustice with the most severe consequences: when women’s knowledge of their own labour experiences is systematically discredited, the resulting epistemic wrong can lead to catastrophic harm. Moreover, all these are examples of avoidable harm, and so to improve understanding of how and why avoidable harm occurs, appreciating the impact of testimonial injustice is vital.

As a result of failing to properly assess a patient’s condition, such a failure of care might also constitute a recognizable legal wrong, a possibility that carries damaging ramifications for individuals and organizations accused (as well as the victim). Harm caused by a breach of the professional standard of care might amount to negligence, with consequent damages paid to a successful claimant. Space prevents analysis of the challenges and impact of pursuing a negligence claim either for claimants or indeed the impact and resourcing burden placed on defendants, but it is worth noting that^130^ damages paid out diminish the funds available to provide good levels of staffing and other resources, which is likely to contribute to some of the problems, including the epistemic harms, identified in the reports we have studied. If the patient dies and the conduct involved is deemed to be grossly negligent, there may be criminal liability for gross negligence manslaughter.^131^ Police investigations were launched following the Ockenden review,^132^ and so criminal prosecutions might eventually follow. It is, however, questionable how far blaming individuals for gross negligence manslaughter serves to address the type of organizational failures discussed here, as Ost has observed in her analysis of the conviction of Dr Bawa Garba.^133^ Organizational criminal liability for corporate manslaughter would seem better suited, but the evidential burdens of corporate manslaughter make it difficult to prove.^134^ The Corporate Manslaughter and Corporate Homicide Act 2007 has been described by Menis as a ‘fiction of criminalisation’ that pays only lip service to public demands for justice,^135^ so the chances of organizational criminal liability seem low. What is clear, however, is that epistemic injustice is a wrong from which these legal consequences flow.

These possible legal consequences highlight the serious wrong done to the victim, and might be seen to provide opportunities to address the injustice perpetrated. But all too often, justice will not be served because of the challenges of bringing a civil claim and/or the high evidential threshold for criminal liability. Crucially, understanding how epistemic injustice ultimately enables such harms could lead to more effective improvements in care, and a reduced clinical negligence bill which would in turn increase NHS resources to enable improved care. By addressing testimonial injustice directly, healthcare systems could reduce the risk of the catastrophic outcomes that may eventually trigger legal consequences.

### B. The consequential impact on patient trust

A significant secondary consequence of epistemic injustice is the erosion of trust in HCPs and the organizations within which they work. Trust in healthcare incorporates the expectation that healthcare professionals will respect patient autonomy by listening to the patient’s account of their condition and symptoms to provide appropriate, beneficial care. When patient testimony is ignored or belittled, patients will understandably feel that they have been wronged as a primary harm. When that wrong also results in poor physical or mental outcomes, which might be consequential harms, such as that seen in the scandals we have discussed above, patient trust in the individual/s and the organization responsible for that harm will be severely damaged if not destroyed. The patient is likely to feel betrayed, and that betrayal and the direct erosion of trust is only the start. Mistrust is contagious. The family and friends of the person harmed might also experience a decline in trust in HCPs and organizations. When racism is present, entire communities lose trust.^136^ And when patient safety failings become a public matter because of a particularly tragic case or a scandal that is widely reported and discussed (such as the examples discussed above), societal fear of medical error and consequent mistrust of HCPs may also grow. On that basis, high-profile scandals may be particularly damaging to societal levels of trust in medicine.

Wolfensberger and Wrigley’s exploration of trust is especially useful for our account of testimonial injustice.^137^ They suggest that uncertainty and risk are the most important features of trust. How the patient perceives the *competence* of the physician and the patient’s sense of the doctor’s *commitment* to a patient-oriented approach are crucial. When a patient’s account of their condition and the evidence about what is happening to their body is ignored, the patient will have good reason to doubt both the competence and the commitment of the HCP concerned. Where medicine is concerned, trust is relational and is a justifiable expectation (not a belief), founded on a cognitive mental state. Patients are constantly engaged in actively assessing those who care for them, rather than blindly trusting them from the outset,^138^ and so when important information provided by the patient is disregarded, the patient may reasonably doubt professional competence and will certainly question whether that professional is committed to a patient-oriented approach.

The decline of trust damages the therapeutic relationship: patients may avoid seeking help, or will be more likely to question the advice and treatment offered, less likely to believe that the physician is acting in their best interests and less likely to follow the treatment plan, thus becoming a more ‘difficult patient’ in the eyes of the HCP and perpetuating the cycle of epistemic injustice. Evidence suggests that when women seeking healthcare are dismissed and not taken seriously, they may experience trauma and consequent fear of accessing necessary health services.^139^ This may obviously have dire consequences for the patient if they decide not to seek care or not to follow a treatment plan.

Also, when testimonial injustice is present, the very notion of shared decision-making and an ‘honest two-way transfer of knowledge’ becomes impossible. If patient testimony is ignored, the patient is unable to share responsibility. Instead, they are expected to simply accept professional opinion in circumstances where they have every reason to question the competence and commitment of the HCP. What unites both dimensions of harm considered in this section (the legal consequences explored in Subsection A and the erosion of trust explored in subsection B) is the failure to listen. It is testimonial injustice, the systematic dismissal of women’s accounts of their own bodies and experiences, that creates the conditions for both the legal wrongs and the relational harms we have described. Understanding this is essential if healthcare systems and their regulators are to address not merely the symptoms of poor care, but its root cause.

## VI. Regulating to overcome epistemic injustice

Our analysis reveals widespread and repeated harms to women caused to them in their healthcare as a result, we argue, of testimonial injustice. The question then arises as to what is needed from a regulatory perspective to prevent such avoidable harms.^140^ Here, we turn to the potential for appropriately formulated patient safety regulation to play a key role in avoiding harms to patients by highlighting and preventing the risk of epistemic injustice.^141^ In order to do this, we argue, it must also avoid the risk of perpetuating testimonial injustice itself.

The scale of iatrogenic harm, ‘*preventable* adverse events *resulting from medical therapy*’,^142^ has long been a driver of harm prevention efforts in patient safety regulation across the globe.^143^ Learning lessons from such events and the implications for patients of failing to do so play a crucial role in such efforts in England.^144^ It has been increasingly emphasized in numerous public inquiry reports,^145^ and forms a central strand of the recommendations of those reports examined in this article.^146^ Government continues to accept that learning lessons from patient harm is critical to prevention efforts,^147^ with a resulting prevalence of this imperative throughout patient safety regulation strategies and frameworks.

This commitment to learning from patient harm has recently taken on renewed urgency in maternity care, where the systemic failures discussed herein have prompted the most recent high-level government response—a national investigation of maternity and newborn care in 14 hospital trusts.^148^ The investigation, led by Baroness Amos, aims to put the voices of mothers and families at the heart of the work and is expected to report directly to the Health and Social Care Secretary, Wes Streeting, in the spring of 2026.^149^ In her interim report, she commented that, ‘nothing prepared me for the scale of unacceptable care that women and families have received, and continue to receive, the tragic consequences for their babies, and the impact on their mental, physical and emotional wellbeing’.^150^ Alongside this investigation, Streeting also announced a National Maternity and Neonatal Task Force, which he will chair personally, to help drive improvement across the NHS.^151^ As a priority, the Task Force will be responsible for answering ‘how can we ensure that women and their partners are always listened to when they raise concerns about their pregnancy or labour?’.^152^ He further announced a package of measures intended to increase accountability across the board and invoke the necessary cultural change, though further details on this are not yet clear.^153^

While these recent announcements demonstrate continued government commitment to learning from patient safety failures, the effectiveness of such responses depends critically on their form, remit, and how they are implemented. A full account and analysis of all extant patient safety regulations and policies in England is not possible within this article,^154^ and so here we focus on two retrospective regulatory frameworks of particular relevance to women’s healthcare in England: the Maternity and Newborn Safety Investigation Programme (MSNI) and the Patient Safety Incident Response Framework (PSIRF). Each arises out of a particular patient safety strategy and has a specific regulatory body that applies a distinctive investigatory approach. Our focus on these frameworks allows us to illustrate that, while regulatory efforts are being made in patient safety regulation to respond to inquiry findings that patients are not being listened to, not basing such responses on the *reasons* why patients are not heard in their care, dooms them to failure and, worse, to the risk of replicating testimonial injustice themselves.

MNSI’s purpose is ‘to provide independent, standardised and family focused investigations of maternity cases for families’, analysing key trends to ensure system-wide learning.^155^ It was established as one of the responses to the *Safer Maternity Care* national action plan, which sets out to achieve the National Maternity Safety Ambition of halving stillbirth and neonatal and maternal death rates and birth-related brain injuries by 2025.^156^ The Care Quality Commission (Maternity and Newborn Safety Investigation Programme) Directions 2023 (the 2023 Directions) have from 1 October 2023, required the CQC to carry out maternity investigations under MSNI.

The 2023 Directions include duties on the CQC to consider concerns raised by mothers or family members in relation to their care or the care of their baby^157^ and to consult with and seek evidence from them as part of their investigation.^158^ Drafts of investigation reports, including a draft summary of the facts, must be given to the mother or her representatives or family, as well as the care providers concerned,^159^ giving them a reasonable time to consider it,^160^ and then taking into account any comments and making revisions where necessary.^161^ These steps suggest an acceptance that including patients as information providers in safety investigations can lead to a more fully informed understanding of the causes of harm. In addition, MSNI’s investigators have been provided with training sessions by Birthrights to help them to understand human rights and systemic racism in maternity care and potentially their own investigations, and to ‘instil a deeper appreciation for the lived realities of individuals who may face unique challenges within the healthcare system’.^162^ However, two problems remain that undermine MSNI’s likelihood of preventing patient harm.

First, the regulation itself fails to require investigators explicitly to explore whether patients’ treatment has been affected by the prejudices underlying epistemic injustice in healthcare. Secondly, the qualifying criteria for cases that fall within the remit of the 2023 Directions and MSNI’s oversight are limited to those involving a baby or a direct or indirect maternal death.^163^ An eligible baby within the scheme has 37 or more completed weeks of gestation, and either died during birth (intrapartum stillbirth)^164^ or within the first week of life,^165^ or experienced a severe brain injury in the first week of life.^166^ Furthermore, it must be established that none of these outcomes is the result of a congenital or genetic abnormality.^167^ MSNI therefore covers important groups of patients who are evidently at risk of serious harm in their care. However, the strict eligibility criteria mean that many cases covered in the reports that we examined above would fall outside the jurisdiction of the MNSI inspectors who have been trained to be alert to at least some of the prejudices we have highlighted.

Those patients who are not included within MSNI’s remit may fall within the purview of the PSIRF, which was piloted under England’s 2019 NHS Patient Safety Strategy, with a focus on understanding *how* incidents leading to patient harm happen. The Strategy was updated in 2021 to include an increased focus on addressing patient safety inequalities,^168^ and all NHS trusts were required to implement the PSIRF from September 2022.^169^ The PSIRF acknowledges that NHS organizations have found it challenging to conduct good quality investigations which contribute to learning and thus reduce the risk of harm to patients.^170^ It therefore provides guidance on how acute, ambulance, mental health, and community healthcare services should respond to a patient safety incident,^171^ and prioritises ‘compassionate engagement and involvement of those affected by patient safety incidents’.^172^

A focus on the PSIRF allows us to highlight a further potential regulatory flaw, which is the risk that epistemic injustice will be perpetuated in *investigations* into the causes of patient harm. One of the PSIRF’s core elements following a patient safety incident is engaging and involving patients, families, and staff.^173^ This aims to ensure compassion and listening to all those involved. The Patient Safety Incident Response Standards^174^ state that engagement leads should communicate and engage with those involved in a positive and compassionate way^175^ and ‘listen and hear the distress of others in a measured and supportive way’.^176^ Those affected should be ‘meaningfully involved’.^177^ However, there is nothing within the framework to highlight to investigators the potential for epistemic exclusion of patients within the inquiry, or to help them to understand the potential contributions of epistemic injustice to the patient’s experience of their healthcare. While some patients will be listened to under the PSIRF, there is no reason to suspect that those conducting investigations within its purview are exempt from the prejudices patients can be subject to in the course of their care. Without an explicit embedding of a knowledge of the existence and harms of epistemic injustice, there is a risk that patients will not be seen as epistemically credible by investigators, and thus not truly heard in the sense of contributing to a full account of the causes and effects of their poor experiences.

While the MNSI and PSIRF are fundamentally important regulatory provisions in attempting to learn from harm and improve future care for women and persons of female biology, they are only two out of a plethora of patient safety initiatives.^178^ Here, they serve as examples of the ways in which regulation that is not based on a knowledge and understanding of epistemic injustice in this context will fail to that ensure lessons are learned, meaning that the epistemic harms already experienced by patients may be perpetuated.

The regulatory approaches noted here indicate an understanding at the policy level that learning from error is a key patient safety tool because, if there is no understanding of how harm is caused, prevention efforts cannot be targeted effectively. They also illustrate a commitment to implementing patient safety regulation, which ostensibly responds to inquiry findings that patients should be listened to, by involving them in PSIRF or MSNI investigations. However, current approaches are destined to *hear* but not *listen* due to the absence of a crucial element—an understanding of testimonial injustice as a critical risk factor in women’s healthcare.

## VII. Conclusion

As we have demonstrated above, extensive evidence of testimonial injustice permeates all areas of women’s healthcare. This epistemic marginalization directly correlates with missed opportunities for learning, inadequate investigations, and poor care outcomes, sometimes with devastating consequences. It is becoming increasingly recognised that understanding the intersecting factors that lead to disparities in healthcare is essential to effectively tackling and overcoming them.^179^ Patients consistently report that they want to be listened to, both about the care they would like to receive and regarding the management of adverse events. Indeed, in 2016, the Better Births review noted that ‘women wanted to be listened to about what they want for themselves and their baby, and to be taken seriously when they raise concerns’.^180^ The maternity context is not alone in this recognition.^181^

Yet, the policy response to this issue has to be more than a recognition that HCPs need to listen to women.^182^ Crucially, our concern is that typical approaches to addressing these failures—primarily recommendations calling for the establishment of a culture of compassion and kindness in care or repeated assertions that patients should be listened to—are inadequate because they fail to address the underlying epistemic structures that position women as unreliable narrators of their own experiences. Such approaches assume that failures of this nature are isolated either to specific healthcare contexts, for example, maternity care, or isolated to specific hospitals or personnel, and this fundamentally ignores the structural patterns that routinely devalue women’s knowledge and experiences across the whole sector of women’s healthcare.

It is important to emphasize that this article is *not* a call for regulation to implement mechanisms of assigning blame to individual HCPs, as it is evident that this is detrimental to learning in the aftermath of error.^183^ Rather, this article calls for regulatory and educational responses grounded in a nuanced understanding of epistemic injustice, and further work will be required to explore what such interventions may involve. Such interventions likely must extend beyond regulatory reform alone. Medical culture and the ‘hidden curriculum’^184^—the informal transmission of values, assumptions, and stereotypes that occurs alongside formal clinical training—play a significant role in perpetuating the epistemic harms we have identified by embedding unexamined biases about women’s credibility in new generations of clinicians. Addressing testimonial injustice in women’s healthcare, therefore requires cultural and educational change within medical institutions. Both the healthcare system and its regulators must recognize and actively counter the systematic devaluation of women’s testimonial knowledge. Only by acknowledging these epistemic wrongs can we begin to address the issues that systematically undermine women’s health outcomes. Without this fundamental reframing, regulatory responses risk perpetuating the very prejudices they seek to address.

## Footnotes

1 See NHS, ‘Women’s Health’ (n.d) <https://www.nhs.uk/womens-health/> accessed 20 June 2025.

2 We acknowledge that trans men and non-binary people also access these healthcare services. We use gendered language in this article to discuss specific prejudices against and stereotypes of women that we suggest are relevant to the way that they are perceived and treated making them particularly vulnerable to epistemic injustice. However, trans-identifying and non-binary people may also be particularly vulnerable to epistemic injustice owing to prejudice against their specific identities as trans and non-binary individuals.

3 Department of Health and Social Care, *Women’s Health Strategy for England* (Cm 736, 2022).

4 ibid 2.

5 ibid 6.

6 J Cumberlege, ‘First Do No Harm – The Report of the Independent Medicine and Medical Devices Safety Review’ (the Cumberlege Report) (2020) <https://www.immdsreview.org.uk/downloads/IMMDSReview_Web.pdf> accessed 20 June 2025.

7 D Ockenden, ‘Findings, Conclusions and Essential Actions from the Independent Review of Maternity Services at The Shrewsbury and Telford Hospital NHS Trust’ (2022) <https://www.ockendenmaternityreview.org.uk/wp-content/uploads/2022/03/FINAL_INDEPENDENT_MATERNITY_REVIEW_OF_MATERNITY_SERVICES_REPORT.pdf> accessed 23 June 2025.

8 Birthrights, ‘Systemic Racism Not Broken Bodies – An Inquiry into Racial Injustice and Human Rights in UK Maternity Care’ (2022) <https://www.birthrights.org.uk/wp-content/uploads/2022/05/Birthrights-inquiry-systemic-racism-May-22-web-1.pdf> accessed 23 June 2025.

9 M Peter and R Wheeler, ‘The Black Maternity Experiences Survey A Nationwide Study of Black Women’s Experiences of Maternity Services in the United Kingdom – Five X More’ (2022) <https://fivexmore.org/blackmereport> accessed 20/06/2025. And see their 2025 report at < https://fivexmore.org/black-maternity-experiences-report-2025> accessed 23 February 26.

10 B Kirkup, ‘Reading the Signals – Maternity and Neonatal Services in East Kent – The Report of the Independent Investigation’ HC 681 (2022) <https://assets.publishing.service.gov.uk/media/634fb083e90e0731a5423408/reading-the-signals-maternity-and-neonatal-services-in-east-kent_the-report-of-the-independent-investigation_print-ready.pdf> accessed 20 June 2025.

11 Welsh Parliament, Health and Social Care Committee, ‘Unheard: Women’s Journey Through Gynaecological Cancer’ (2023) <https://senedd.wales/media/5ztjb5fm/cr-ld16197-e.pdf> accessed 20 June 2025.

12 K Thomas, ‘Listen to Mums: Ending the Postcode Lottery on Perinatal Care A report by The All-Party Parliamentary Group on Birth Trauma’ (2024) <https://www.theo-clarke.org.uk/sites/www.theo-clarke.org.uk/files/2024-05/Birth%20Trauma%20Inquiry%20Report%20for%20Publication_May13_2024.pdf> accessed 20 June 2025.

13 Cumberlege (n 6) ii, 2, 17–21, 140; Ockenden (n 7) 14, 107, 116, 133, 193; Birthrights (n 8) 37, 49, 52–55, 66–67; Peter and Wheeler (n 9) 23–24; Kirkup (n 10) 1–8, 20–22, 36–37, 39–52, 62–67, 159–60; Welsh Parliament (n 11) 17–23; Thomas (n 12) 8, 14–15, 32, 65–66.

14 A number of the reports we considered also highlighted the challenges for healthcare staff in attempting to provide safe care for women, and their own struggles to be heard where they felt that this was not happening. This important issue is acknowledged here, but space does not permit a full consideration of it.

15 See eg Cumberlege (n 6) which details the chronic pain pelvic mesh patients have experienced. See Ockenden (n 7), at para 2.4 where reported cases of maternal and neonatal harm are stated to include: cases of stillbirth, neonatal death, maternal death, hypoxic ischaemic encephalopathy (HIE) (grades 2 and 3) and other complications in mothers and newborn babies. See Kirkup (n 10) 6–8 where an illustrative case of harm to a patient’s wellbeing resulting from a failure to listen and to respond compassionately is outlined. See also Welsh Parliament (n 11), at para 15–16 where the story of Judith, a woman whose terrible pain and concerns that her cancer had returned were ignored. By the time she was diagnosed, Judith’s cancer was incurable, and she died shortly after her video evidence was shown in Committee.

16 Department of Health and Social Care (n 3).

17 HC Deb 30 January 2025, vol 761, col 428.

18 Department of Health and Social Care (n 3) 8.

19 Women and Equalities Committee, *Women’s Reproductive Health Conditions* (HC 24-25, 337-I).

20 See eg Department of Health, *The Report of the Public Inquiry into Children’s Heart Surgery at the Bristol Royal Infirmary 1984-1995: Learning from Bristol* (Cmd 5207(I), 2001); R Francis, ‘The Mid Staffordshire NHS Foundation Trust. Public Inquiry Chaired by Robert Francis QC. Report of the Mid Staffordshire NHS Foundation Trust Public Inquiry’, 3 vols, (2013) <https://www.gov.uk/government/publications/independent-inquiry-into-care-provided-by-mid-staffordshire-nhs-foundation-trust-january-2001-to-march-2009> accessed 20 July 2025; National Advisory Group on the Safety of Patients in England, ‘A Promise to Learn – A Commitment to Act: Improving the Safety of Patients in England’ (2013) <https://assets.publishing.service.gov.uk/government/uploads/system/uploads/attachment_data/file/226703/Berwick_Report.pdf> accessed 20 July 2025.

21 M Fricker, *Epistemic Injustice: Power and the Ethics of Knowing* (OUP 2007) 1 (author’s own emphasis).

22 V May, ‘Speaking into the Void? Intersectionality Critiques and Epistemic Backlash’ (2014) 29 Hypatia 94; G Polhaus Jr, ‘Varieties of Epistemic Injustice’ in IJ Kidd, J Medina and G Pohlhaus Jr, *The Routledge Handbook of Epistemic Injustice* (Routledge 2017) 37–40.

23 Polhaus Jr, ibid,37.

24 CK Gott, *Experience, Identity and Epistemic Injustice within Ireland’s Magdalene Laundries* (Bloomsbury 2023) 22.

25 May (n 22) 98; Polhaus Jr (n 22) 37; J Medina, ‘Feminism and Epistemic Injustice’ in KQ Hall and Ásta (eds), *The Oxford Handbook of Feminist Philosophy* (OUP 2021) 409.

26 Fricker (n 21).

27 ibid 22.

28 ibid 136.

29 Fricker (n 21) 17.

30 ibid 130–31.

31 ibid.

32 ibid 154–59, 169–70.

33 ibid 152–61 (s 7.2).

34 ibid 149–52.

35 ibid; building on this concept for a discussion of rape myths and domestic abuse as hermeneutical injustices see also K Jenkins, ‘Rape Myths and Domestic Abuse as Hermeneutical Injustices’ (2017) 34 Journal of Applied Philosophy 191.

36 ibid; H Carel and IJ Kidd, ‘Epistemic Injustice in Medicine and Healthcare’ in IJ Kidd, J Medina and G Polhaus Jr (eds), *The Routledge Handbook of Epistemic Injustice* (Routledgen 2017).

37 This focus is due to space constraints and should not be taken to indicate that hermeneutical injustice is not a feature of healthcare in this context.

38 Fricker (n 21) 35.

39 ibid 23–27.

40 Kathy’s story was originally reported in 2015 by the then active news site ThinkProgress, see ThinkProgress, ‘When Gender Stereotypes Become a Serious Hazard to Women’s Health’ <https://archive.thinkprogress.org/when-gender-stereotypes-become-a-serious-hazard-to-womens-health-f1f130a5e79/> accessed 20 June 2025. It also featured in C Criado Perez, *Invisible Women – Exposing Data Bias in a World Designed for Men* (Chatto and Windus 2019) 223–24.

41 ibid.

42 ibid.

43 ibid.

44 Fricker (n 21) 43–44.

45 ibid 43.

46 ibid 44.

47 ibid.

48 ibid 46–48.

49 ibid.

50 ibid 47–48.

51 L Merone and others, ‘"I just want to feel safe going to a doctor" Experiences of female patients with chronic conditions in Australia’ (2022) 3 Women’s Health Reports: ‘I Just Want to Feel Safe Going to a Doctor’: Experiences of Female Patients with Chronic Conditions in Australia | Women’s Health Reports.

52 See, eg N Tuana, ‘The Speculum of Ignorance: The Women’s Health Movement and Epistemologies of Ignorance’ (2006) 21 Hypatia 1; H Carel and IJ Kidd, ‘Epistemic Injustice in Healthcare: A Philosophical Analysis’ (2014) 17 Medicine, Health Care and Philosophy 529; L Freeman, ‘Confronting Diminished Epistemic Privilege and Epistemic Injustice in Pregnancy by Challenging a “Panoptics of the Womb”‘(2015) 40 Journal of Medicine and Philosophy 44; IJ Kidd and H Carel, ‘Epistemic Injustice and Illness’ (2017) 34 Journal of Applied Philosophy 172; C Blease, H Carel and K Geraghty, ‘Epistemic Injustice in Healthcare Encounters: Evidence from Chronic Fatigue Syndrome’ (2017) 43 Journal of Medical Ethics 549; EA Byrne, ‘Striking the Balance with Epistemic Injustice in Healthcare: The Case of Chronic Fatigue Syndrome/Myalgic Encephalomyelitis’ (2020) 23 Medicine, Health Care and Philosophy’ 371; R Lakeman, ‘Epistemic Injustice and the Mental Health Service User’ (2010) 19 International Journal of Mental Health Nursing 151; P Crichton, H Carel and IJ Kidd, ‘Epistemic Injustice and Psychiatry’ (2017) 41 BJ Psych Bull 65; AP Scrutton, ‘Epistemic Injustice and Mental Illness’ in IJ Kidd, J Medina and G Polhaus Jr (eds), *The Routledge Handbook of Epistemic Injustice* (Routledge 2017); L Bortolotti (ed), *Epistemic Injustice in Mental Healthcare – Recognising Agency and Promoting Virtues Across the Life Span* (Springer Nature 2025); Y Peled, ‘Language Barriers and Epistemic Injustice in Healthcare Settings’ (2018) 32 Bioethics 360; A Younas, ‘Epistemic Injustice in Health Care Professionals and Male Breast Cancer Patients Encounters’ (2021) 31 Ethics & Behaviour 451.

53 Carel and Kidd, ‘Epistemic Injustice in Medicine and Healthcare’ (n 36) 336.

54 ibid.

55 ibid.

56 ibid.

57 ibid; LT Rosen, ‘Mapping out Epistemic Justice in the Clinical Space: Using Narrative Techniques to Affirm Patients as Knowers’ (2021) 16 Philosophy, Ethics and Humanities in Medicine 1; Kidd and Carel, ‘Epistemic Injustice and Illness’ (n 52); Scrutton (n 52).

58 L Nimmon and T Stenfors-Hayes, ‘The “Handling” of Power in the Physician–patient Encounter: Perceptions from Experienced Physicians’ (2016) 16 BMC Medical Education 114; See also M Foucault, *The Birth of the Clin* (AM Sheridan-Smith tr, Routledge 2003); JP Bishop, ‘Revisiting Foucault’ (2009) 34 Journal of Medicine and Philosophy 323.

59 NHS, ‘NHS Long Term Plan – 1. Empowering People’ (n.d.) <https://www.longtermplan.nhs.uk/online-version/chapter-5-digitally-enabled-care-will-go-mainstream-across-the-nhs/1-empowering-people/> accessed 20 May 2025; P Bravo and others, ‘Conceptualising Patient Empowerment: A Mixed Methods Study’ (2015) 15 BMC Health Services Research 252.

60 Bravo and others, ibid; M Boudioni, S McLaren and G Lister, ‘A Critical Analysis of National Policies, Systems, and Structures of Patient Empowerment in England and Greece’ (2017) 11 Patient Preference and Adherence 1657.

61 Nimmon and Stenfors-Hayes (n 58) 7.

62 Carel and Kidd, ‘Epistemic Injustice in Healthcare: A Philosophical Analysis’ (n 52) 530.

63 ibid 536.

64 Kidd and Carel, ‘Epistemic Injustice and Illness’ (n 52) 175.

65 Examples of chronic somatic conditions include chronic fatigue syndrome, fibromyalgia, and irritable bowel syndrome.

66 ibid.

67 Rosen (n 57).

68 Kidd and Carel, ‘Epistemic Injustice and Illness’ (n 52) 177.

69 ibid 177–78.

70 L O’Donovan, ‘Listen[ing] to Mums: Epistemic Injustice in UK Maternity Care’ (*The Polyphony*, 14 February 2025) <https://thepolyphony.org/2025/02/14/listening-to-mums/> accessed 17 January 2026.

71 ibid.

72 R Chung, ‘Structural Health Vulnerability: Health Inequalities, Structural and Epistemic Injustice’ (2021) 52 Journal of Social Philosophy 201.

73 Kidd and Carel, ‘Epistemic Injustice and Illness’ (n 52) 178.

74 eg on women’s pain not being believed see Ipsos, ‘Hysterical Health – Unpicking the Cultural Beliefs that Shape Women’s Healthcare’ (2023) <https://www.ipsos.com/en-uk/hysterical-health-unpicking-cultural-beliefs-that-shape-womens-healthcare> accessed 20 June 2025.

75 R Auchmuty, ‘Feminist Approaches to Sexuality and Law Scholarship’ (2015) 15 Legal Information Management 4, 6 (author’s own emphasis).

76 E Cleghorn, ‘Medical Myths about Gender Roles go back to Ancient Greece. Women are Still Paying the Price Today’ *TIME* (New York, 17 June 2021) <https://time.com/6074224/gender-medicine-history/> accessed 20 May 2025; E Cleghorn, *Unwell Women – A Journal through Medicine and Myth in a Man-Made World* (Weidenfeld & Nicolson 2021).

77 ibid.

78 ibid.

79 JB Bonnard, ‘Male and Female Bodies According to Ancient Greek Physicians’ (LE Doherty and VS Cuchet trs) (2013) 37 Women, Gender, History 1.

80 Cleghorn (n 76).

81 ibid.

82 C Tasca and others, ‘Women and Hysteria in the History of Mental Health’ (2012) 19 Clinical Practice & Epidemiology in Mental Health 110; M Trimble and EH Reynolds, ‘A Brief History of Hysteria: From the Ancient to the Modern’ (2016) 139 Handbook of Clinical Neurology 3.

83 ibid 116.

84 EC Romanis and others, ‘Reviewing the Womb’ (2021) 47 JME 820.

85 L Merone and others, ‘Sex Inequalities in Medical Research: A Systematic Scoping Review of the Literature’ (2022) 3 Women’s Health Reports 49, 49–50.

86 eg it takes on average eight years for patients to receive a diagnosis of endometriosis. See All Party Parliamentary Group on Endometriosis, ‘Endometriosis in the UK – Time for Change’ (2020) <https://www.endometriosis-uk.org/sites/default/files/files/Endometriosis%20APPG%20Report%20Oct%202020.pdf> accessed 20 July 2025.

87 Cumberlege (n 6) 20.

88 TRANS vaginal tape–obturator.

89 Cumberlege (n 6), 151.

90 Welsh Parliament, Health and Social Care Committee (n 11), 17.

91 Thomas (n 12) 14.

92 Kirkup (n 10), 44.

93 Ockenden (n 7).

94 Ipsos (n 74); DE Hoffman and AJ Tarzian, ‘The Girl Who Cried Pain: A Bias against Women in the Treatment of Pain’ (2001) 29 Journal of Law, Medicine & Ethics 13; Criado-Perez (n 40).

95 Hoffman and Tarzian, ibid; A Samulowitz and others, ‘“Brave Men” and “Emotional Women”: A Theory Guided Literature Review on Gender Bias in Health Care and Gendered Norms towards Patients with Chronic Pain’ (2018) 1 Pain Research and Management 1; G Jackson, *Pain and Prejudice: A Call to Arms for Women and their Bodies* (Piatkus 2019); Criado-Perez (n 40); Cleghorn (n 76).

96 ibid.

97 Birthrights (n 8) 58, 63.

98 Hoffman and Tarzian (n 94); Ipsos (74) 11; Cleghorn (n 76); L Zhang and others, ‘Gender Biases in Estimation of Others Pain’ (2021) 22 Journal of Pain 1048.

99 Cleghorn (n 76); Jackson (n 95); L Appignanesi, *Mad, Bad and Sad: A History of Women and the Mind Doctors from 1800 to the Present* (Hachette 2008); JM Usher, *The Madness of Women - Myth and Experience* (Routledge 2011) 812; A Scull, *Hysteria – The Disturbing History* (OUP 2012).

100 Women and Equalities Committee, *Black Maternal Health* (2022–23, HC 94-III); Z Khan, ‘Ethnic Health Inequalities in the UK’s Maternity Services: A Systematic Literature Review’ (2021) 29 British Journal of Midwifery 100.

101 R Samra and O Hankivsky, ‘Adopting an Intersectionality Framework to Address Power and Equity in Medicine’ (2021) 397 Lancet 857.

102 A Felker, R. Patel, R Kotnis, S Kenyon, and M. Knight (eds), on behalf of MBRRACE-UK, *Saving Lives, Improving Mothers’ Care Compiled Report - Lessons learned to inform maternity care from the UK and Ireland Confidential Enquiries into Maternal Deaths and Morbidity 2021-23*, (2025) Oxford: National Perinatal Epidemiology Unit, University of Oxford < https://www.npeu.ox.ac.uk/mbrrace-uk/reports/maternal-reports/maternal-report-2021-2023> accessed 23 February 2026. Allison Felker, Roshni Patel, Rohit Kotnis, Sara Kenyon, and Marian Knight (eds) on behalf of MBRRACE-UK. Saving Lives, Improving Mothers’ Care Compiled Report - Lessons learned to inform maternity care from the UK and Ireland Confidential Enquiries into Maternal Deaths and Morbidity 2020-22. Oxford: National Perinatal Epidemiology Unit, University of Oxford (October 2024) <https://www.npeu.ox.ac.uk/mbrrace-uk/reports/maternal-reports/maternal-report-2020-2022> accessed 7 July 2025.

103 M Knight and others, on behalf of MBRRACE-UK, ‘A National Cohort Study and Confidential Enquiry to Investigate Ethnic Disparities in Maternal Mortality’ (2021) 43 eClinicalMedicine 101237.

104 ibid 12.

105 ibid.

106 Peter and Wheeler (n 9) 23.

107 Birthrights (n 8) 63.

108 ibid 22, 52, 58–59, 63.

109 ibid 47.

110 Peter and Wheeler (n 9) 23.

111 ibid.

112 Birthrights (n 8) 53.

113 T Jones and KJ Norwood, ‘Aggressive Encounters & White Fragility: Deconstructing the Trope of the Angry Black Woman’ (2017) 102 Iowa Law Review 2017.

114 Women and Equalities Committee (n 100) 11.

115 Knight and others (n 103), 13; Birthrights (n 8); Women and Equalities Committee (n 100).

116 PA Braveman and others, ‘Systemic and Structural Racism: Definitions, Examples, Health Damages, and Approaches to Dismantling’ (2022) Health Affairs 41 171–78.

117 PA Braveman and others, ‘Systemic and Structural Racism: Definitions, Examples, Health Damages, and Approaches to Dismantling’ (2022) Health Affairs 41 171–78.

118 Cumberlege (n 6) 7.

119 Department of Health and Social Care and The Rt Hon Wes Streeting MP, ‘Health and Social Care Secretary Speech at RCOG World Congress’ (23 June 2025) <https://www.gov.uk/government/speeches/health-and-social-care-secretary-speech-at-rcog-world-congress> accessed 01 July 2025.

120 Department for Health and Social Care (n 3).

121 ibid 2, 15.

122 ibid 15.

123 ibid.

124 Women and Equalities Committee (n 19) 21.

125 ibid.

126 ibid.

127 Kirkup (n 10) 43.

128 Birthrights (n 8) 54.

129 Ockenden (n 7) 104.

130 Annual Statistics from NHS Resolution: Annual statistics—NHS Resolution And see; NHS facing £27bn bill for maternity failings in England | The Independent.

131 C Dyer, ‘First Case of Corporate Manslaughter against NHS Collapses’ (2016) 352 BMJ i585.

132 A Patel, ‘Police Reviewing more than 100 Maternity Cases’ *BBC News* (21 November 2024) <https://www.bbc.co.uk/news/articles/cpwre51d22yo> accessed 7 July 2025.

133 S Ost, ‘Drs Bramhall and Bawa-Garba and the Rightful Domain of the Criminal Law’ (2019) 45 Journal of Medical Ethics 3, 151–55.

134 A Patel, ‘Police Reviewing more than 100 Maternity Cases’ *BBC News* (21 November 2024) <https://www.bbc.co.uk/news/articles/cpwre51d22yo> accessed 7 July 2025.

135 S Menis, ‘The Fiction of the Criminalisation of Corporate Killing’ (2017) 81 Journal of Criminal Law 467–77.

136 See, eg S Hamad H. Bradby, B. Maina-Ahlberg et al.and others, ‘Racism in Healthcare: A Scoping Review’ (2022) 22 BMC Public Health 988.

137 M Wolfensberger and A Wrigley, *Trust in Medicine* (CUP 2019), chapter 11. The authors suggest that such betrayal has a moral component. Other work is broadly relevant to trust in medicine but less so to testimonial injustice, see, eg Z Fritz and R Holton, ‘Too Much Medicine: Not Enough Trust?’ (2019) 45 Journal of Medical Ethics 31; CC Clark, ‘Trust in Medicine’ (2002) 27 Journal of Medicine and Philosophy 11.

138 Wolfensberger and Wrigley, ibid 120.

139 L Merone and others, ‘“I just want to feel safe going to a doctor” Experiences of Female Patients with Chronic Conditions in Australia’ (2022) 3 Women’s Health Reports: ‘I Just Want to Feel Safe Going to a Doctor’: Experiences of Female Patients with Chronic Conditions in Australia | Women’s Health Reports

140 Patient Safety Learning, ‘Mind the Implementation Gap: The Persistence of Avoidable Harm in the NHS’ (2022) <https://d2z1laakrytay6.cloudfront.net/Mindtheimplementationgap_ThepersistenceofavoidableharmintheNHS_2022-04-07-121554_vhao.pdf> accessed 7 July 2025. For more on the efficacy or otherwise of public inquiries, see National Audit Office, *Investigation into Government-Funded Inquiries* (HC 2017–19, 836); Institute for Government, ‘How Public Inquiries can Lead to Change’ (2017) <https://www.instituteforgovernment.org.uk/sites/default/files/publications/Public Inquiries %28final%29.pdf> accessed 7 July 2025; Statutory Inquiries Committee, *Public Inquiries: Enhancing Public Trust* (HL 2024-25, 9).

141 We focus on mechanisms within England to do so, but our arguments apply to patient safety regulation more generally. This is also not to say that other initiatives such as combatting the transmission of established prejudices in clinical training should not also be undertaken, see H Carel and IJ Kidd, ‘Epistemic Injustice in Medicine and Healthcare’ in IJ Kidd, J Medina and G Polhaus Jr (eds), *The Routledge Handbook of Epistemic Injustice* (Routledge 2017)) and C Lawrence and others, ‘The Hidden Curricula of Medical Education: A Scoping Review’ (2018) Academic Medicine 93 648–56.

142 A Merry and W Brookbanks, *Merry and McCall Smith’s Error’s, Medicine and the Law* (2nd edn, CUP 2017) 55 (emphasis in original).

143 S Devaney, ‘Errors, Medicine and the Law: Learning Lessons about how to Learn from Mistakes in Healthcare’ in S Fovargue and C Purshouse, *Leading Works in Health Law and Ethics* (Routledge 2024).

144 Department of Health, ‘An Organisation with a Memory: Report of an Expert Group on Learning from Adverse Events in the NHS Chaired by the Chief Medical Officer’ (2000) <https://psnet.ahrq.gov/issue/organisation-memory-report-expert-group-learning-adverse-events-nhs-chaired-chief-medical> accessed 7 July 2025.

145 Department of Health, *The Report of the Public Inquiry into Children’s Heart Surgery at the Bristol Royal Infirmary 1984-1995: Learning from Bristol* (Cm 5207, 2001); National Advisory Group on the Safety of Patients in England, ‘A Promise to Learn – A Commitment to Act: Improving the Safety of Patients in England’ (2013) <https://assets.publishing.service.gov.uk/government/uploads/system/uploads/attachment_data/file/226703/Berwick_Report.pdf> accessed 7 July 2025; R Francis, ‘Freedom to Speak Up. An Independent Review into Creating an Open and Honest Reporting Culture in the NHS’ (2015) <http://freedomtospeakup.org.uk/wp-content/uploads/2014/07/F2SU_web.pdf> accessed 7 July 2025, and B Kirkup, ‘The Report of the Morecombe Bay Investigation’ (2015) <https://assets.publishing.service.gov.uk/media/5a7f3d7240f0b62305b85efb/47487_MBI_Accessible_v0.1.pdf> accessed 7 July 2025.

146 Cumberlege (n 6) 49, 184; Ockenden (n 7) 5–6, 39–41, 54; Birthrights (n 8) 91; Peter and Wheeler (n 96) 4, 36, 40; Kirkup (n 10) 162–63; Thomas (n 12) 66.

147 Department of Health, *Learning not Blaming: The government response to the Freedom to Speak Up Consultation, the Public Administration Select Committee Report ‘Investigating Clinical Incidents in the NHS’, and the Morecambe Bay Investigation* (Cm 9113, 2015).

148 Department of Health and Social Care, ‘14 NHS trusts the focus of national maternity investigation’ (15 September 2025) <https://www.gov.uk/government/news/14-nhs-trusts-the-focus-of-national-maternity-investigation> accessed 1 September 2025.

149 ibid.

150 Amos V, Independent Investigation into Maternity and Neonatal Services in England—Reflections and Initial Impressions (December 202 <https://www.matneoinv.org.uk/updates/independent-investigation-into-maternity-and-neonatal-services-in-england-reflections-and-initial-impressions/> accessed 23 February 2026.

151 Department of Health and Social Care (n 119).

152 ibid.

153 ibid.

154 For non-maternity patient safety initiatives, see details of all initiatives at NHS England, ‘Patient Safety’ <https://www.england.nhs.uk/patient-safety/> accessed 7 July 2025. In the maternity sector see eg NHS England, ‘Three Year Delivery Plan for Maternity and Neonatal Services’ (2023) <https://www.england.nhs.uk/wp-content/uploads/2023/03/B1915-three-year-delivery-plan-for-maternity-and-neonatal-services-march-2023.pdf> accessed 7 July 2025; the initiatives outlined in E Parkin, B Balogun and R Harker, ‘House of Commons Library Research Briefing: Quality and safety of maternity care (England)’ (6 March 2024) <https://researchbriefings.files.parliament.uk/documents/CBP-9815/CBP-9815.pdf> accessed 7 July 2025; and those contained in Department of Health and Social Care, ‘Government Response to Reading the Signals: Maternity and Neonatal Services in East Kent–the report of the Independent Investigation’ (August 2023) <https://www.gov.uk/government/publications/maternity-and-neonatal-services-in-east-kent-report-government-response> accessed 7 July 2025.

155 Maternity and Newborn Safety Investigations, ‘Our Governance’ (n.d) <https://www.mnsi.org.uk/about/our-governance/> accessed 7 July 2025.

156 Department of Health, ‘Safer Maternity Care: Next Steps Towards the National Maternity Ambition’ (October 2016) <https://assets.publishing.service.gov.uk/media/5a80efa3ed915d74e33fd3d7/Safer_Maternity_Care_action_plan.pdf> accessed 7 July 2025. Note that the Plan is unlikely to achieve the Ambition within that timeframe–see B Balogun and others, *Quality and Safety of Maternity Care* (England) (2025) <https://commonslibrary.parliament.uk/research-briefings/cbp-9815/> accessed 7 July 2025.

157 Direction 2 (4)(d). Direction 2 (4)(e) requires the CQC to consider concerns raised by anyone involved in the care under investigation, and anyone else it thinks appropriate.

158 Direction 5.

159 Direction 4(2).

160 Direction 4(3).

161 Direction 4(4).

162 MNSI, ‘Inspiring Inclusion and Empowering MNSI Investigators’ (2024) <https://www.mnsi.org.uk/news/inspiring-inclusion-and-empowering-mnsi-investigators/> accessed 7 July 2025.

163 para 3(1) of the Schedule sets out that maternal deaths fall within these directions if occurring ‘within 42 days of the end of the pregnancy from any cause related to or aggravated by the pregnancy or its management, but not from accidental or incidental causes’.

164 para 2(1)(a).

165 para 2(1)(b).

166 para 2(2).

167 para 2(1)(b).

168 NHS, ‘NHS Patient Safety Strategy: 2021 Update’ (2021) <https://www.england.nhs.uk/wp-content/uploads/2021/02/B0225-NHS-Patient-Safety-Strategy-update-Feb-2021-Final-v2.pdf> accessed 8 July 2025.

169 NHS Providers, ‘On the Day Briefing - Patient Safety Incidence Response Framework’ (2022) <https://nhsproviders.org/media/694034/on-the-day-briefing-patient-safety-incident-response-framework.pdf> accessed 8 July 2025.

170 NHS England and NHS Improvement, ‘Patient Safety Incident Response Framework 2020: An Introductory Framework for Implementation by Nationally Appointed Early Adopters’ (March 2020) <https://www.england.nhs.uk/wp-content/uploads/2020/08/200312_Introductory_version_of_Patient_Safety_Incident_Response_Framework_FINAL.pdf > accessed 8 July 2025.

171 Defined as ‘unintended or unexpected incidents which could have or did lead to harm for one or more patients receiving healthcare’, ibid 13.

172 HSIB, Learn Together and NHS England, ‘Patient Safety Incident Response Framework Supporting Guidance: Engaging and involving patients, families and staff following a patient safety incident’ (2022) <https://www.england.nhs.uk/wp-content/uploads/2022/08/B1465-2.-Engaging-and-involving…-v1-FINAL.pdf> accessed 8 July 2025.

173 ibid.

174 NHS England, ‘Patient Safety Incident Response Standards’ (2024) <https://www.england.nhs.uk/long-read/patient-safety-incident-response-standards/> accessed 8 July 2025.

175 ibid, para 9.1

176 ibid, para 9.2

177 ibid, para 13.

178 See the sources listed at n 147.

179 MA Bohren and others, ‘Towards a Better Tomorrow: Addressing Intersectional Gender Power Relations to Eradicate Inequities in Maternal Health’ (2024) 67 eClinicalMedicine 102180.

180 NHS England, ‘National Maternity Review’ (n.d.) <https://www.england.nhs.uk/mat-transformation/implementing-better-births/mat-review/> accessed 8 July 2025. See also J Jomeen and M Redshaw, ‘Ethnic Minority Women’s Experience of Maternity Services in England’ (2013) 18 Ethn Health 280 for a similar finding.

181 Department of Health and Social Care (n 13); Department of Health and Social Care, ‘Results of the “Women’s Health – Let’s Talk About It” Survey’ (2022) <https://www.gov.uk/government/calls-for-evidence/womens-health-strategy-call-for-evidence/outcome/results-of-the-womens-health-lets-talk-about-it-survey> accessed 8 August 2025.

182 O’Donovan (n 70).

183 J Reason, ‘Human Error: Models and Management’ (2000) 320 The BMJ 768; NHS Resolution, ‘Being Fair: Supporting a Just and Learning Culture for Staff and Patients following incidents in the NHS’ (2019) <https://resolution.nhs.uk/wp-content/uploads/2019/07/NHS-Resolution-Being-Fair-Report-2.pdf> accessed 8 July 2025.

184 M Brown and others, ‘Exploring the Perceptions Of Senior Medical Students on Gender and Pain: A Qualitative Study of the Interplay between Formal and Hidden Curricula’ BMJ Open 2024; 14:e080420.

